# Dopamine D1 receptor in the NAc shell is involved in delayed emergence from isoflurane anesthesia in aged mice

**DOI:** 10.1002/brb3.1913

**Published:** 2020-10-22

**Authors:** Yi Zhang, Huan Gui, Lang Hu, Chengxi Li, Jie Zhang, Xiaoli Liang

**Affiliations:** ^1^ Department of Anesthesiology the Second Affiliated Hospital of Zunyi Medical University Zunyi China; ^2^ Guizhou Key Laboratory of Anesthesia and Organ Protection Zunyi Medical College Zunyi China; ^3^ School of Anesthesiology Zunyi Medical University Zunyi China

**Keywords:** aging, delayed emergence, dopamine D1 receptor, general anesthesia, nucleus accumbens

## Abstract

**Background:**

Delayed emergence after general anesthesia tends to occur in the elderly population, but the mechanism remains unclear. Apart from age‐related pharmacokinetic changes, the aging‐induced structural and functional alterations in the arousal‐promoting neural substrates should be considered. The nucleus accumbens (NAc) is a crucial arousal‐related nucleus, in which activating medium spiny neurons (MSNs) expressing dopamine D1 receptor (D1R) could facilitate the arousal from natural sleep. Meanwhile, the dopaminergic systems decline with aging in multiple brain regions. However, whether the age‐related decline in D1R in the NAc shell attenuates its arousal‐promoting capacity from general anesthesia remains to be elucidated.

**Methods:**

We first verified the delayed emergence from isoflurane anesthesia and examined the corresponding changes of electroencephalogram (EEG) power in aged mice. In turn, the arousal‐modulating capacity of D1R was characterized in the young and aged cohorts by microinjection of D1R agonist/antagonist into the NAc shell. Furthermore, to address the possible mechanism responsible for the attenuated arousal‐modulating capacity of the aged NAc, the expression of D1R in the NAc shell was measured and compared between young and aged mice.

**Results:**

Our data indicated that compared with young mice, the emergence time in aged mice was notably longer, while EEG power in δ band (1‐4Hz) was significantly higher and power in β band (12‐25Hz) was lower. Activating or inhibiting D1R in the NAc shell by microinjection D1R agonist/antagonist promoted or delayed the emergence process in young mice. Nevertheless, this modulation capacity of D1R in the NAc shell declined in aged mice, respectively. Meanwhile, downregulation of D1R expression in the NAc shell was detected in the aged brain.

**Conclusion:**

Together, these results suggest that aging attenuates the arousal‐modulating capacity of D1R in the NAc shell probably through downregulation of D1R expression therein, which may provide a potential explanation and a therapeutic target for increased sensitivity to anesthetics in the elderly patients.

## Graphical abstract

Emergence time from isoflurane anesthesia was delayed and cortical EEG power was altered during the emergence process in aged mice. Activating or inhibiting D1R in the NAc shell promoted or delayed the emergence process in terms of behavior and EEG power. Nevertheless, the modulation capacity of D1R in the NAc shell declined with age, probably due to the age‐related downregulation of D1R in the NAc shell. These data may provide a potential pharmacodynamical explanation and a therapeutic target for delayed emergence from general anesthesia and increased sensitivity to anesthetics in elderly patients.

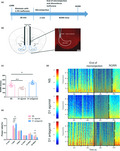

## 1 INTRODUCTION

Delayed emergence from general anesthesia is defined as the state that patients fail to regain consciousness and make thoughtful respond to commands or stimuli 30–60 min after general anesthesia (Ellis et al., 2017). Growing age is a critical risk factor for delayed emergence not only due to the gradual decline in hepatorenal function but also due to pharmacodynamic alterations (Anastasian, Ornstein, & Heyer, 2009). It has been demonstrated that concentrations of isoflurane and sevoflurane required to maintain the value of bispectral index (BIS) below 50 (MAC_BIS50_) decrease with the advance in age (Matsuura et al., 2009), suggesting that sensitivity of aged brain to anesthetics is remarkably increased. Nevertheless, the neural correlates and potential mechanisms responsible for the changes are still poorly understood.

The dopaminergic pathway in the brain is implicated in the sleep–wake cycle (Oishi & Lazarus, 2017; Qiu et al., 2019), of which ventral tegmental area (VTA) plays a key role in the emergence process of general anesthesia (Taylor et al., 2016). The nucleus accumbens (NAc) in the ventral striatum primarily consists of medium spiny neurons (MSNs) expressing substantial dopamine D1 receptor (D1R) and D2 receptor (D2R) and receives substantial dopaminergic projections from the VTA (Soares‐Cunha et al., 2016). It has been shown that optogenetic activation of NAc D1R‐expressing neurons results in a transition from natural sleep to wakefulness (Luo et al., 2018). As well, systemic administration of D1R agonist can shorten the time to emergence from isoflurane anesthesia (Taylor et al., 2013). Thus, it is conceivable that D1R in the NAc plays a crucial role in the emergence from general anesthesia. On the other hand, age‐related declines occur in the structure and function of the cerebral dopaminergic system, including dopamine (DA) neuronal loss, reduction of DA synthesis, and downregulation of DA receptors (Rollo, 2009). However, it remains sparsely documented whether these age‐related declines, in particular, the alterations of D1R in the NAc shell attenuate the arousal‐promoting capacity of the NAc and thus prolong the time to emergence from general anesthesia in the elderly subject. Here, we first determined the difference in the emergence time from isoflurane anesthesia and electroencephalogram (EEG) power spectrum between young and aged mice. In turn, the arousal‐modulating capacity of D1R in the NAc shell was characterized in the young and aged cohorts by microinjection of D1R agonist/antagonist into the NAc shell. Furthermore, the expression of D1R in the NAc was detected and compared to address the potential mechanisms responsible for the attenuated arousal‐modulating capacity of the aged NAc.

## 2 MATERIAL AND METHODS

### 2.1 Animals

All experiments were approved by the Animal Care and Use Committees of Zunyi Medical University, Guizhou, China, and carried out in accordance with the “Guide for the care and use of laboratory animals” in China (No. 14,924, 2001). Young C57BL/6 mice (5–8 months old, male, weight: 28–40 g) and aged C57BL/6 mice (18–22 months old, male, weight: 30–42 g) were used in this study. Mice were housed in standard cages in rooms with a 12‐hr reverse light–dark cycle. The temperature was controlled to 23 ± 2°C in a relative humidity of 55 ± 2%. Food and water were available ad libitum to mice. To minimize the effect of circadian rhythms, all behavioral and EEG experiments were performed between 4:00 p.m. and 10:00 p.m.

### 2.2 Stereotaxic surgery for EEG electrode placement and microinjection

Mice were anesthetized with 50mg/kg pentobarbital intraperitoneally and placed on a stereotaxic frame (RWD Life Science, Shenzhen, China). Lidocaine (1%) was subcutaneously injected for local analgesia. For cortical EEG recording, an electrode was placed into the prefrontal cortex (PFC, AP: +1.8 mm, ML: 0.0 mm, DV: −2.5 mm) according to stereotactic coordinates from the mice brain atlas (Paxinos & Franklin, 2004) and a reference electrode was placed on the skull. For local microinjection, a 26‐gauge bilateral guide cannulas (O.D.0.34 mm × I.D.0.2 mm, length: 4.5 mm; RWD Life Science, Shenzhen, China) was implanted into the unilateral NAc shell (AP + 1.54 mm, ML ± 0.7 mm, and DV‐4.50 mm), and fixed to the skull with two stainless steel screws and dental acrylic. All animals were individually housed and allowed to recover for one week before the experiment.

### 2.3 Behavioral tests and EEG recording

To compare induction and emergence time from isoflurane anesthesia between young and aged mice (*n* = 8), animals connected to flexible counterbalanced cables for EEG recording were placed in an induction chamber prefilled with 1.4% isoflurane (1MAC; Lunan Pharma Co., Ltd.) and 1L O2 + 1L air/min carrier gas. The time to loss of righting reflex (LORR) was recorded. EEG signals during this process were amplified by a Model 3,000 High‐Gain AC/DC Amplifier (1 Hz–3 kHz, gain: 1,000 ×; A‐M systems, Inc.) and recorded by Spike 2 software package (Cambridge Electronic Design). Isoflurane anesthesia was maintained for 30 min at the concentration of 1.4% which was continuously monitored by a gas analyzer. The temperature in the chamber was kept at 37°C, and the floor was covered with soda lime. Then, isoflurane was flushed via a disposable charcoal filter placed in a fume hood. The time from isoflurane concentration fell to 0 to the recovery of righting reflex (RORR), which was defined as RORR time, and EEG during this process were also recorded.

In the microinjection experiment, 18 young mice and 18 aged mice were assigned to 3 groups, respectively (*n* = 6). Anesthesia was induced and maintained as mentioned above. D1R agonist chloro‐APB hydrobromide and D1R antagonist SCH‐23390 (Sigma‐Aldrich) were dissolved in 10% dimethyl sulfoxide (DMSO) in distilled water prior to microinjection. At 28th minute, chloro‐APB hydrobromide (5 μg/0.5 μl), SCH‐23390 (5 μg/0.5 μl), and 0.5 μl saline were injected into the NAc shell of mice in D1 agonist group, D1R antagonist group, and normal saline (NS) control group at a rate of 0.25 μl/min via a microsyringe pump (Legato® 130; KD Scientific)., respectively. The doses of D1 agonist/antagonist were determined on the basis of our preliminary experiment, as shown in Figure S1. After administration, the injection cannula was kept in the guide cannula for 1min to prevent the agents from refluxing back up the cannula rather than diffusing into the tissue. Meanwhile, isoflurane was discontinued and waste gas in the chamber was suctioned off. The RORR time and EEG signals throughout the process were recorded. The schematic timeline for microinjection experiment was shown in Figure 2a.

### 2.4 Histological verification

To verify whether guide cannulas were accurately placed into the NAc shell, at the end of experiments, the mice were anesthetized with sodium pentobarbital (60 mg/kg, i.p.) and transcardially perfused with 0.01 M PBS followed by 4% PFA. The brain was removed and fixed in 4% PFA for 24 hr, followed by incubation in 30% sucrose in PBS for 24 hr at 4°C. According to the mice brain atlas (Paxinos & Franklin, 2004), a 6‐mm section containing the NAc was cut by razor blades and mounted with anterior side facing up on a freezing block at −80°C. Then, 40‐μm slices were cut and placed in plates filled with PBS. Brain slices were mounted to glass slides, and cannula tracks were observed under a stereomicroscope (BX51W1‐IR7; Olympus). The subjects with incorrect position of cannula tip were excluded. A representative histological verification for cannula location is shown in Figure 2b.

### 2.5 EEG power spectral analysis

The EEG data analysis was completed using Spike 2 software package. To analyze power spectrum, cortical EEGs from the end of microinjection to RORR in each group were extracted and filtered to delta (1–4 Hz), theta (4–8 Hz), alpha (8–12 Hz), beta (12–25 Hz), and gamma (25–60 Hz) bands, respectively. Power spectrum of each band was normalized to the total power in individual animal, and the relative ratios were compared between different groups.

### 2.6 Western blot analysis for D1R expression in the NAc

Tissues of the NAc shell in young and aged mice were collected from a 2‐mm coronal section using a 1‐mm microdissection punch. Samples were homogenized in 3% (w/v) Tris‐HCl buffer (pH 7.5, 50 mM at 4°C) added with proteinase inhibitors and then centrifuged at 20,000 × g for 10 min at 4℃. Protein concentrations were tested using Bradford reagent (Bio‐Rad). Equal amounts of samples were loaded and separated on a 12% SDS–polyacrylamide electrophoresis gel. Gels were transferred to polyvinylidene fluoride membrane at 30 V for 16 hr at 4°C. Membranes were blocked with 0.2% BSA/10 mM Tris, 150 mM NaCl, and 1% Tween‐20 (TBST) prior to incubation with primary antibody (1:500; Abcam) overnight at 4°C. After washing, membranes were then incubated with HRP‐conjugated goat anti‐mouse secondary antibodies (1:10,000; Abcam) for 2 hr at room temperature. The band intensity was detected and measured using the ChemiDoc™ MP Imaging System (Bio‐Rad). Levels of target protein were normalized to β‐actin.

### 2.7 Statistical analysis

Statistical analysis was carried out using GraphPad Prism software package version 6.0 (GraphPad Software Inc.). The normality of data was determined by the one‐sample Kolmogorov–Smirnov test. Differences between aged and young mice were detected using Student's *t* test. Differences between the three groups were tested by one‐way ANOVAs with Tukey's multiple comparison test. Changes in the EEG power spectrum were analyzed by two‐way ANOVA followed by a Bonferroni post hoc test. The significance level for all tests was set at *p* < .05.

## 3 RESULTS

### 3.1 Aging delayed emergence time and altered cortical EEG power during the emergence process

**Figure 1:**
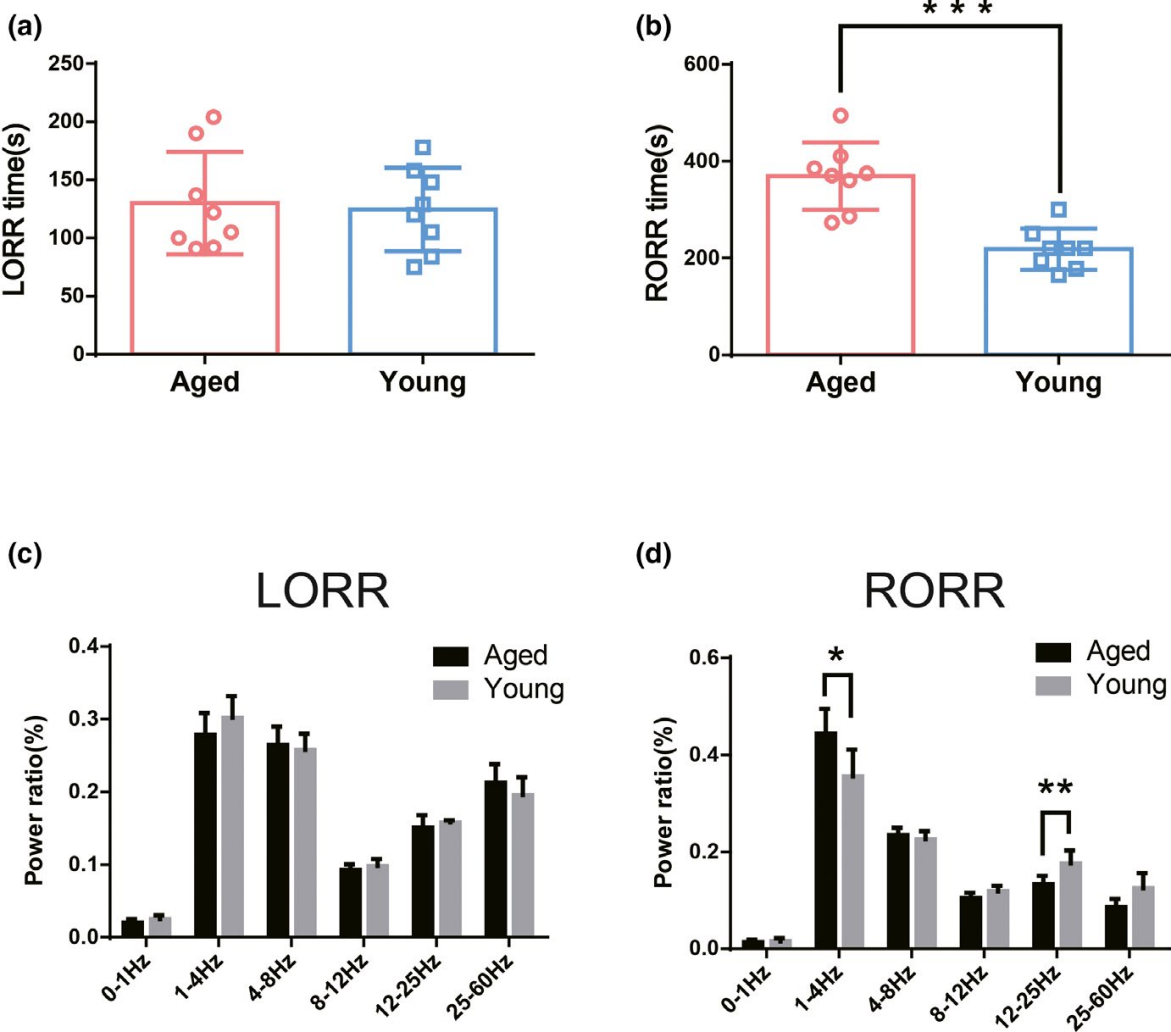
Differences in the time and EEG during induction and emergence period between aged and young mice (*n* = 8). (a) There was no significant difference in induction time between the two groups. (b) Emergence time was prolonged in the aged mice. *** *p* < .001. (c) There was no statistical difference in each band of power spectrum between aged and young mice during the induction process. (d) Power in the δ band was increased and power in the β band was reduced in aged mice during the emergence process. * *p* < .05, **p < .01

There was no significant difference in the LORR time between aged and young mice (130.1 ± 44.21 vs 124.6 ± 35.95, *t* = 0.273, *p* = .789), as shown in Figure 1a. RORR time in aged mice was notably longer than that in young mice (369.1 ± 42.6 vs 218.5 ± 69.47, *t* = 5.228, *p < *.001), as shown in Figure 1b. Results from EEG analysis demonstrate that during induction process each band of power spectrum in aged mice did not differ with that in young mice (*p* > .05), whereas during emergence process the power in δ band (1‐4Hz) was significantly higher (*t* = 2.858, *p* = .017) and power in β band (12‐25Hz) was lower (*t* = 3.247, *p* = .009) in aged mice than that in young mice, as shown in Figure 1c,d.

### 3.2 Microinjection of D1R agonist/antagonist into the NAc shell‐regulated emergence from isoflurane anesthesia in young mice

**Figure 2:**
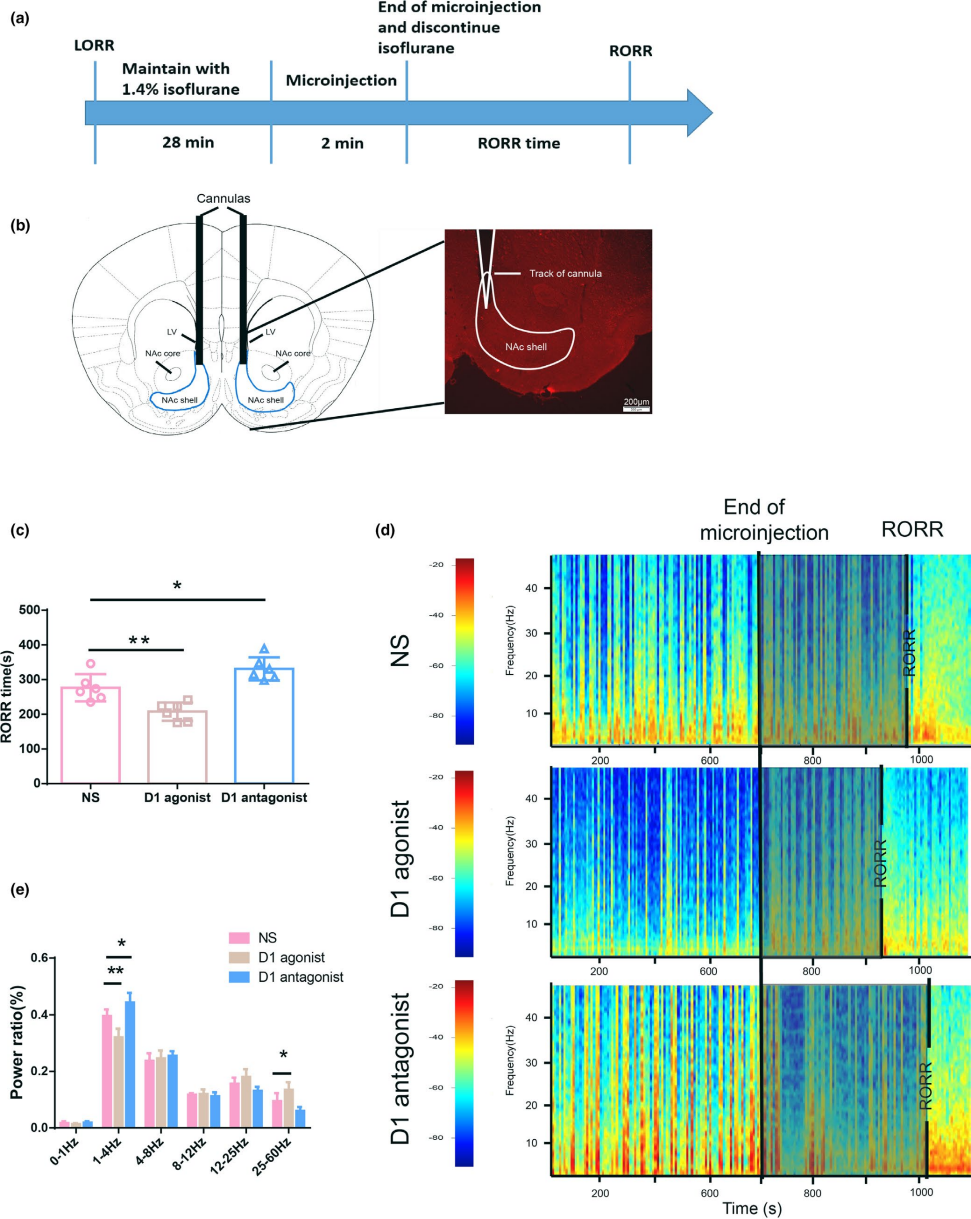
Microinjection of D1 agonist/antagonist into NAc shell modulated the emergence process from isoflurane anesthesia in young mice. (a) Schematic timeline for microinjection experiment. (b) Histological verification of cannula location in the NAc shell. (c) Microinjection of D1R agonist/antagonist into the NAc shell shortened/prolonged the emergence time from isoflurane anesthesia respectively. *n* = 6, * *p* < .05, **p < .01. (d) Representative EEG spectrograms of the emergence process in the three groups. Note that after microinjection of D1R agonist/antagonist, the power spectra were enhanced or inhibited compared with that before microinjection. Also, the gray parts represent the emergence process, which were shortened or prolonged by D1R agonist/antagonist. (e) Microinjection of D1R agonist into the NAc shell reduced δ power and enhanced γ power, while microinjection of D1R antagonist increased δ power, as compared to NS controls. * *p* < .05, **p < .01

Compared with the NS controls, bilateral microinjection of D1R agonist into the NAc shell significantly reduced the emergence time from isoflurane anesthesia, whereas microinjection of D1R antagonist into NAc shell notably prolonged emergence time (207.7 ± 26.49s for D1R agonist, 331.2 ± 33.39s for D1R antagonist, and 276.3 ± 39.21s for NS, *n* = 6 per group, *F*_(2, 15)_ = 20.55, *p* < .001) (Figure 2c). Meanwhile, activating or inhibiting D1R in the NAc shell boosted or attenuated the arousal in EEG, demonstrating a reduced δ band and an increased γ band in the D1R agonist group, as well as an enhanced δ band in D1R antagonist group comparing with NS controls (*n* = 6, *F*_(10, 90)_=15.7, *p* < .001), as shown in Figure 2d,e.

### 3.3 Microinjection of D1R agonist/antagonist into the NAc shell did not alter the emergence process of aged mice

**Figure 3:**
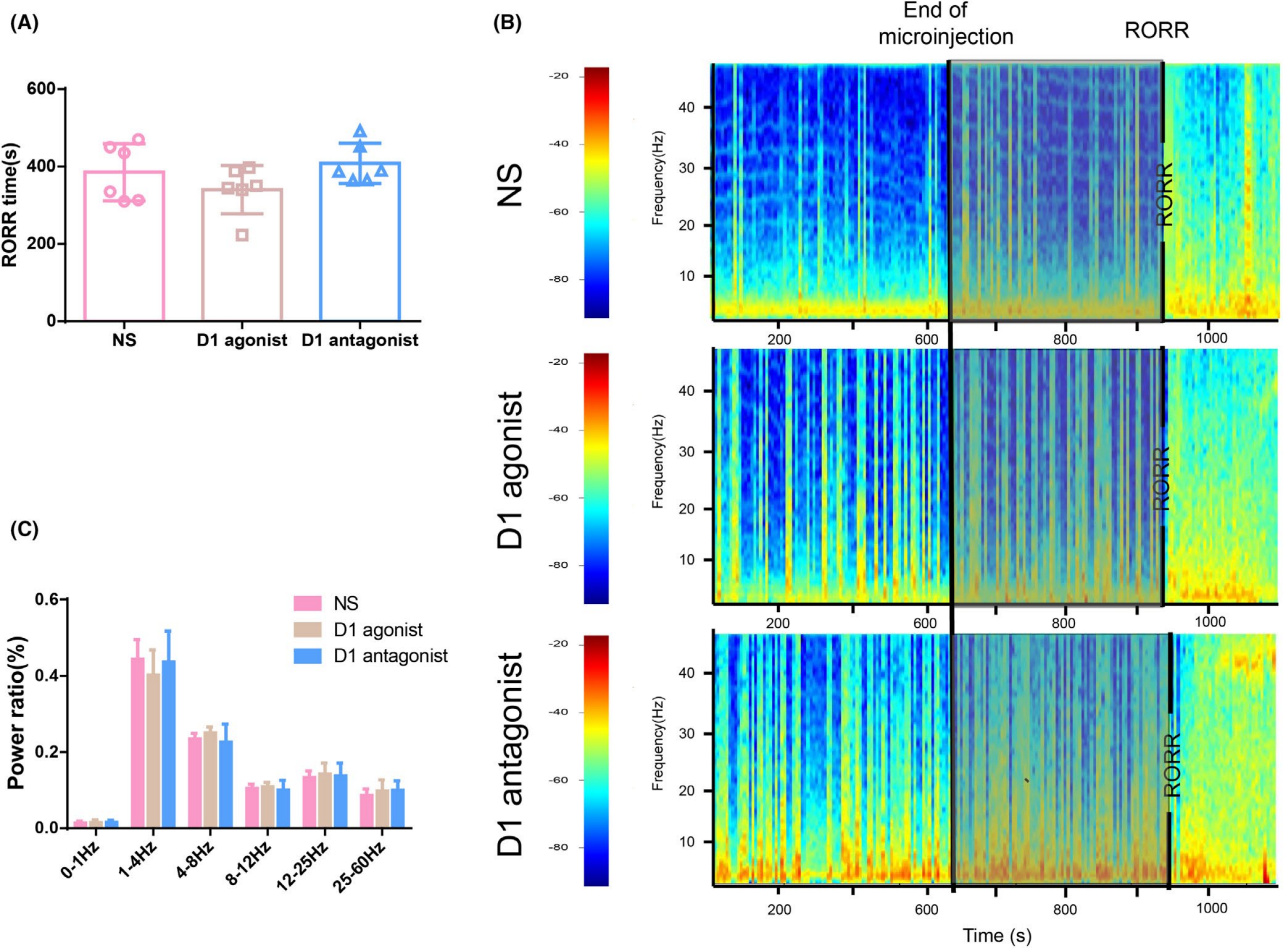
Effect of microinjection of D1R agonist/antagonist into the NAc shell on the emergence process of aged mice. (a) Microinjection of D1R agonist/antagonist into the NAc shell did not alter the emergence time from isoflurane anesthesia. *n* = 6, *p* > .05. (b) Representative EEG spectrograms of emergence process in the three groups. Note that there was no significant alteration in the power spectrum before and after the microinjection of D1R agonist/antagonist. The gray parts represent the emergence process. (c) As compared to NS controls, the microinjection of D1R agonist/ antagonist into the NAc shell did not affect the power in each band

Activating or inhibiting D1R in the NAc shell by microinjection of D1R agonist or D1R antagonist altered neither the emergence time (*n* = 6 per group, *F*_(2,15)_ = 1.792, *p* = .2006) (Figure 3a) nor cortical EEG power in each band (*n* = 6, *F*_(10, 90)_ = 0.753, *p* = .672) (Figure 3b,c) during the emergence process of isoflurane anesthesia in the aged mice.

### 3.4 Age‐related downregulation of D1R in the NAc shell

**Figure 4:**
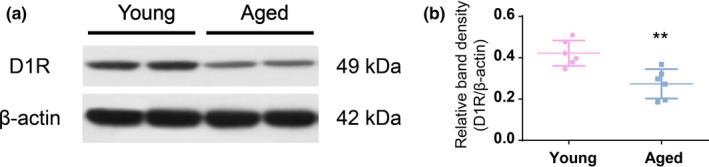
Expression of D1R decreased in the NAc shell of aged mice. (a) Western blot showing D1R protein levels in the NAc shell of two young and two aged mice. (b) Quantitative analysis of Western blot measurement shows the reduced D1R protein level in the NAc shell in aged mice. *n* = 6, Data are expressed as mean ± *SD*, ** *p* < .01

To determine the mechanism responsible for the diminished arousal‐modulating capacity of D1R in the NAc shell, we detected the expression of D1R in both aged and young mice. Compared with young mice, D1R protein level in the NAc shell was prominently lower in aged mice (*n* = 6, *t* = 3.859, *p* = .0032), as shown in Figure 4.

## 4 DISCUSSION

In the present study, we revealed that emergence time from isoflurane anesthesia was delayed and cortical EEG power was altered during the emergence process in aged mice. Activating or inhibiting D1R in the NAc shell promoted or delayed the emergence process in terms of behavior and EEG power. Nevertheless, the modulation capacity of D1R in the NAc shell declined with age, probably due to the age‐related downregulation of D1R in the NAc shell.

A study in rats has reported that aging prolongs emergence after both inhaled isoflurane and intravenous propofol anesthesia, along with lower EEG power and more burst suppression, indicating that the aged brain is more sensitive to anesthetics (Purdon et al., 2015). In line with this study, our data also demonstrate the delayed emergence from isoflurane anesthesia, lower high‐frequency band, and more low‐frequency band during the recovery period in aged mice. This slowing of EEG frequency in aged mice reflects less neural activity in the cortex (Otto, 2008), despite the identical duration and concentration of isoflurane anesthesia to young mice, which suggests that the aged brain is more likely sensitive to anesthetics. Interestingly, aging did not alter behavioral or EEG during the induction process of isoflurane anesthesia. This result may be due to the fact that emergence is not the simply inverse process of induction. Differential neural substrates in the brain are implicated in the induction and emergence of general anesthesia (Friedman et al., 2010). However, the specific mechanism and relevant neural pathways are still required to be further explored.

The NAc consists of two subregions: NAc shell and NAc core, which have different morphology and function. Anatomically, the NAc shell primarily has reciprocal projections with the hypothalamus and the mesencephalon, whereas the NAc core receives projections from the hippocampus and the amygdala (Di Chiara & Bassareo, 2007; Di Ciano & Everitt, 2004; da Cunha et al., 2008). Mounting evidence shows that the sleep–wake pathways in the hypothalamus and mesencephalon are tightly involved with the emergence from general anesthesia (Allada, 2008; Uhrig et al., 2014; Zecharia et al., 2009). Therefore, the NAc shell may be more closely related to general anesthesia than the NAc core. Thus, in this study, we selected the shell as a targeted subregion and verified the arousal‐modulating capacity of the NAc D1R in the emergence from isoflurane anesthesia.

It has been reported that activation of D1R in the NAc by SKF‐38393 can elicit membrane depolarization and inward currents in NAc MSNs, and thus excite D1R‐expressing MSNs (Podda et al., 2010). NAc MSNs send GABAergic projection back to the VTA, preferentially suppressing the interneurons and thus disinhibiting dopamine neurons in the VTA (Creed et al., 2014). It is well characterized that activation of dopamine neurons in the VTA facilitates the emergence from general anesthesia (Solt et al., 2014; Taylor et al., 2016). Together, this mechanism is most likely to underlie the modulation effect of intra‐NAc shell microinjecting D1R agonist/antagonist on the emergence from isoflurane anesthesia in young mice.

However, our results also indicate that this arousal‐modulating capacity of the NAc D1R declines with age. In aged mice, intra‐NAc shell microinjection of D1R agonist/antagonist changed neither the emergence time nor EEG during the process. It has been demonstrated that the normal aging reduces D1R and D2R levels primarily in the frontal cortex and striatum but not the dopamine synthesis capacity (Karrer et al., 2017). Likewise, we found that the expression level of D1R in the NAc shell decreases in the aged mice. Therefore, the age‐related neuroanatomical changes in the cerebral dopaminergic system may contribute to the decline of arousal‐promoting capacity. Nevertheless, differing from our findings, Chemali and colleagues (Chemali et al., 2015) indicate that systemic administration of methylphenidate (an inhibitor of dopamine and norepinephrine transporters) can facilitate the emergence of both propofol and isoflurane anesthesia in aged rats. The reason behind the divergence is that systemic administration of methylphenidate exerts a broader effect on the whole brain rather than exclusively the dopamine receptors in the NAc. The declines of dopaminergic system are not homogeneous in the brain. DA receptors in the frontal cortex and striatum (the NAc is one part of dorsal striatum) seem more susceptible to aging (Suhara et al., 1991; Wang et al., 1998). Therefore, methylphenidate may produce an arousal‐promoting effect via other brain regions in which dopamine receptors are less influenced by aging. Besides, methylphenidate increases noradrenaline levels in the brain as well (Heal et al., 2009), which is known as a key arousal‐promoting neurotransmitter (Brown et al., 2011).

The results reported herein should be considered in light of some limitations. We used exclusively a single concentration of D1R agonist/antagonist referring to relevant literature (Carr et al., 2009), which exerted an appreciable impact in young mice in our preliminary study. Nonetheless, a further concentration gradient of D1R agonist/antagonist would be required especially in aged animals. Additionally, the age‐related alterations in the NAc‐VTA circuit including the loss of DA neurons in the VTA, DA synthesis, and concentration in the NAc should be further elucidated.

In conclusion, the results of the present study indicate that aging attenuates the arousal‐modulating capacity of D1R in the NAc shell probably through downregulation of D1R expression therein, which may provide a potential pharmacodynamical explanation and a therapeutic target for delayed emergence from general anesthesia and increased sensitivity to anesthetics in the elderly patients.

## CONFLICT OF INTEREST

The authors declare no conflict of interests.

## AUTHOR CONTRIBUTIONS

ZY and LXL conceived the idea of this research and drafted the manuscript. GH was responsible for the behavioral tests and staining. HL and ZJ performed the EEG recording and corresponding data analysis. CXL and LXL carried out Western blot and analyzed the data.

### Peer Review

The peer review history for this article is available at https://publons.com/publon/10.1002/brb3.1913.

## Supporting information

Fig S1

## Data Availability

The data that support the findings of this study are available from the corresponding author upon reasonable request.
