# Supplementary figures and images for: Dopamine D1 receptor in the NAc shell is involved in delayed emergence from isoflurane anesthesia in aged mice
Source: Brain Behav. 2020 Oct 22;11(1):e01913. doi: 10.1002/brb3.1913 (PMC7821614; doi:10.1002/brb3.1913)

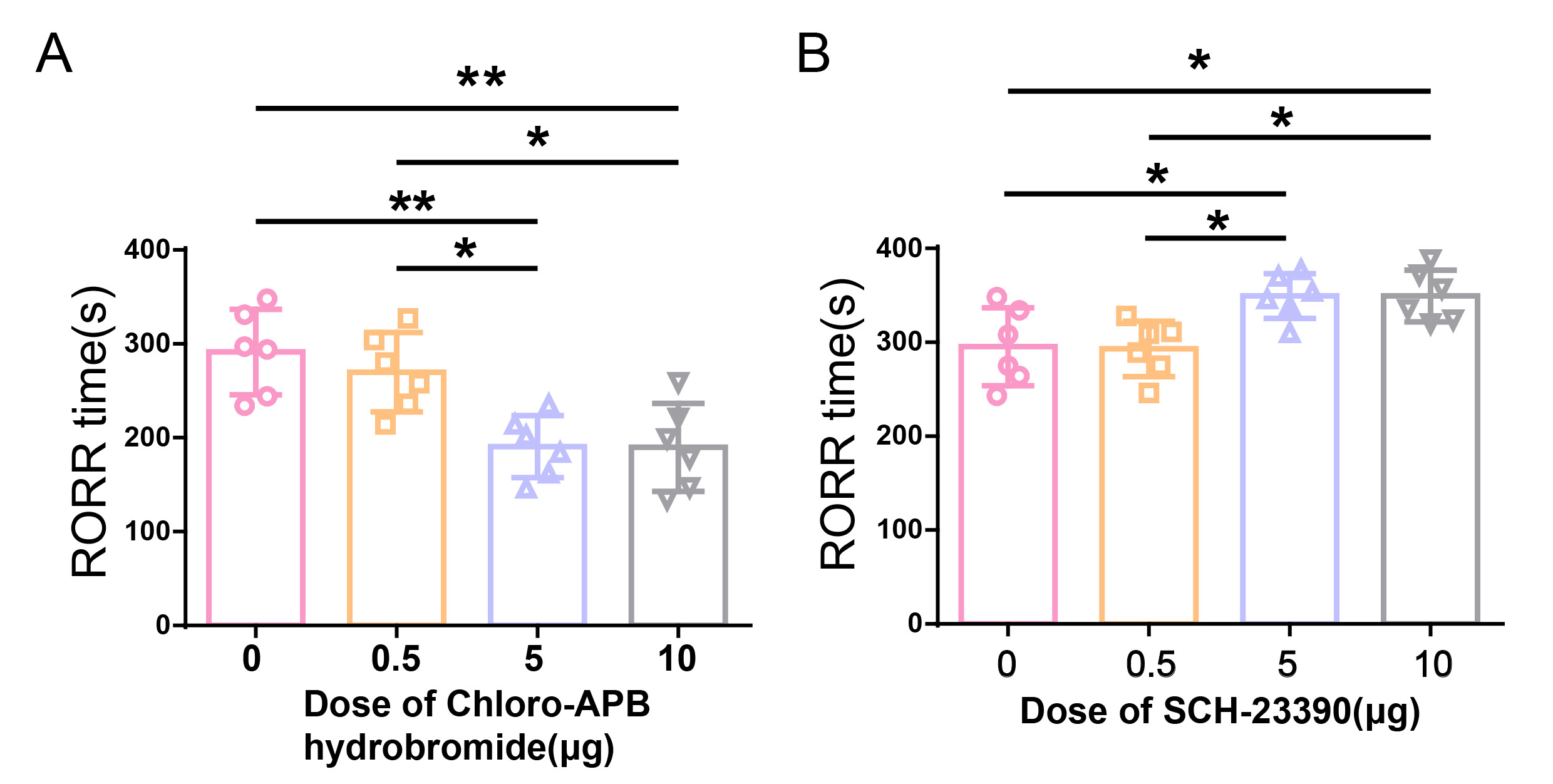

Supplement: Supplementary file 1 — Fig S1 [file BRB3-11-e01913-s001.jpg]
